# Removal of hexavalent chromium by a microbial mat from a mining site under anaerobic conditions

**DOI:** 10.3389/fbioe.2025.1585237

**Published:** 2025-09-12

**Authors:** Mohammad Tariq Ali Khan, Sumaiya Said Al-Siyabi, Hamada E. Ali, Raeid M. M. Abed

**Affiliations:** Biology Department, College of Science, Sultan Qaboos University, Muscat, Oman

**Keywords:** hexavalent chromium, microbial mat, bioreduction, biosorption, facultative anaerobes

## Abstract

Mining activities have contributed to increased contamination of groundwater with hexavalent chromium (Cr(VI)). Microbial mats have been shown to effectively remove Cr(VI) under aerobic conditions, however, their role in Cr(VI) removal under anaerobic conditions remained unexplored. This study investigates the removal of Cr(VI) by a microbial mat collected from a chromite mining site under anaerobic conditions, as well as the underlying mechanism(s). Removal rates of Cr(VI) increased from 0.15 ± 0.01 to 0.77 ± 0.05 mg L^-1^ d^-1^ when the mat was incubated at increasing concentrations from 5 to 50 mg L^-1^, respectively. Biosorption was facilitated by the increased production of extracellular polymeric substances (EPS) after exposure to Cr(VI) with the involvement of functional groups such as metal-O, Cr(VI)-O, PO_4_, C-N, C=O, C-H, Alkyl, and OH-NH_2_. The mat could also reduce Cr(VI) to Cr(III) using chromate reductase enzyme. MiSeq sequencing demonstrated clear shifts in the bacterial community structure in favor of Clostridia and Bacilli at 1 mg L^-1^ Cr(VI), Gammaproteobacteria at 5 mg L^-1^ Cr(VI), and Alphaproteobacteria at the concentrations of 15–50 mg L^-1^ Cr(VI). We conclude that microbial mats contain obligate and facultative anaerobic bacteria that possess the ability to remove Cr(VI) under low fluctuating oxygen levels by biosorption on cell surface and enzymatic reduction to Cr(III).

## 1 Introduction

While numerous studies have concentrated on the aerobic reduction of the highly toxic Cr(VI) to the less toxic and less mobile Cr(III) using bacterial isolates or mixed microbial communities (Das et al., 2021; Acharyya et al., 2023; Alabssawy and Hashem, 2024), there is a growing interest in the anaerobic removal of Cr(VI), driven by its relevance in oxygen-deprived environments such as groundwater and subsurface soils. Under anaerobic conditions, reduction of Cr(VI) to Cr(III) occurs through direct enzymatic pathways and indirect microbially-mediated mechanisms (Wielinga et al., 2001; Thatoi et al., 2014). The direct enzymatic reduction of Cr(VI) involves cytoplasmic chromate reductases (e.g., ChrR), which transfer electrons from organic or inorganic donors to Cr(VI), using it as a terminal electron acceptor (Thatoi et al., 2014). Additionally, a two-steps indirect reduction may take place, e.g., in iron-reducing bacteria such as *Shewanella alga* BrY, where the bacterium reduces other compounds, such as Fe(III), to generate intermediates like Fe(II) that subsequently abiotically reduce Cr(VI) (Wielinga et al., 2001). Environmental factors such as pH, the presence of electron shuttles, and coexisting metals can significantly influence the efficiency of these reduction processes (Narayani and Shetty, 2013). In addition to the process of bioreduction, some anaerobic bacteria have the ability to immobilize Cr(VI) onto their cell surfaces, a process known as bioadsorption. Several bacterial isolates capable of reducing Cr(VI) under anaerobic conditions have been reported (Chen and Gu, 2005; Durán et al., 2018; Huang et al., 2021). For instance, *Pseudomonas dechromaticans* was the first microbe known for Cr(VI) removal under anaerobic conditions and this species was prevalent in early studies (Romanenko and Koren’Kov, 1977; Ehrlich, 1997). Sulfate reducing bacteria such as *Desulfovibrio vulgaris* and *Desulfotomaculum reducens* could reduce Cr(VI) to Cr(III) through two pathways; directly by using hydrogen as an electron donor for enzymatic reduction, or indirectly by producing hydrogen sulfide (H_2_S) that chemically reduced Cr(VI) to Cr(III) (Lovely and Phillips, 1994; Tebo and Obraztsova,1998; Franco et al., 2018). *Shewanella oneidensis*, *Shewanella putrefaciens*, *Exiguobacterium* sp. PY14, *Geobacter sulfurreducens*, *Bacillus cereus*, and *Escherichia coli* were also capable of reducing Cr(VI) under anaerobic conditions (Wang and Shen, 1995; Huang et al., 2023; Cai et al., 2024). The employment of anaerobic microorganisms for Cr(VI) removal is associated with many advantages including: 1) the lower energy requirement, especially for aeration, since these processes do not require oxygen; 2) the use of versatile electron acceptors, other than oxygen, and 3) the simultaneous treatment of other pollutants such as nitrates and sulfates in wastewaters. Therefore, the anaerobic removal of Cr(VI) is considered as a promising approach for the bioremediation of wastewater and industrial effluents, although it remains less studied compared to aerobic processes.

While most previous research on Cr(VI) was conducted using single bacterial isolates, there remains a significant gap in our understanding of Cr(VI) removal by mixed microbial communities. Efficient bioremediation approaches typically involve complex, multispecies consortia where interactions among diverse microorganisms can influence Cr(VI) transformation dynamics. Microbial mats, in particular, play a crucial role in removing pollutants and heavy metals from the environment, by leveraging the physiological versatility of their aerobic and anaerobic microorganisms (Abed et al., 2002; Bender, 2004; Drewniak et al., 2016; Hernandez et al., 2021). It is also well-known that microbial mats produce EPS composed of large negatively charged molecules that can facilitate the binding and sequestration of heavy metals (Nwodo et al., 2012; Mathivanan et al., 2021; Khan et al., 2024). Our studies have recently demonstrated the ability of microbial mats from a chromite mining site to remove 1 mg L^-1^ of Cr(VI) in 7 days under aerobic conditions (Abed et al., 2020). Additionally, aerobic bacteria isolated from the same mats, such as *Bacillus cereus*, *Enterobacter cloacae,* and *Cupriavidus metallidurans*, could tolerate up to 2000 mg L^-1^ Cr(VI) and could remove Cr(VI) at a maximum rate of 100 ± 9 mg L^-1^ d^-1^, through the process of biosorption in EPS and bioreduction with chromate reductase enzyme (Khan et al., 2024). Since microbial mats are known to harbor a diverse community of anaerobic microorganisms, we hypothesize that Cr(VI) removal by these mats can also potentially occur under anaerobic conditions. Indeed, there is a notable scarcity of research related to Cr(VI) by microbial mats, with most existing studies focusing on aerobic processes (Shukla et al., 2012; Abed et al., 2020).

This study investigates the capacity of mixed bacterial communities within natural microbial mats from a chromite mining site in Oman to remove Cr(VI) under anoxic laboratory conditions, with emphasis on the underlying removal mechanism and the shifts in bacterial community composition. By demonstrating Cr(VI) removal through biological and physical processes in complex, native mixed microbial communities, our findings offer new insights into the potential application of microbial mats in the bioremediation of chromium-contaminated aquatic environments with limited oxygen availability.

## 2 Materials and methods

### 2.1 Mat sampling and preparation of reagents

An abandoned chromite mining site in the village of Nakhal, Oman (N 23°24.0079′ E 57°44.5321′) served as the source of water samples and three different types of microbial mats (termed hereafter as Mat A, B, and C). The intact mats were collected in sterile plastic boxes filled with site water, and were brought back immediately to the laboratory in a cool box. Photographs of the sampled mats are provided in Supplementary Figure S1. The physical and chemical parameters (pH, electrical conductivity (EC), oxidation reduction potential (ORP), dissolved oxygen (DO), and temperature) of the site water were measured as previously described (Abed et al., 2020). The site water had a temperature of approximately 25 °C, was alkaline (pH >10), contained low dissolved oxygen (≤1.4 mg/L), and had total Cr concentrations of 45 ± 1 μg L^-1^ (Supplementary Table S1). A stock solution of potassium dichromate (K_2_Cr_2_O_7_) (Cat#104865, Merck, Germany) was prepared at the concentration of 1,000 mg L^-1^, which was then diluted as needed. For EPS isolation, Trichloroacetic acid (TCA) (100% w/v) was prepared by adding 11 g (>99% purity) TCA powder (T6399, Merck, Germany) in 100 mL Milli-Q water. Karnovsky’s fixative buffer was prepared by mixing 10 mL of 5% paraformaldehyde, 10 mL of 50% glutaraldehyde, and sodium-phosphate buffered saline (0.1 M, pH 7.4) with 30 mL of distilled water to make a final volume of 100 mL. The pH was adjusted to 7.2.

### 2.2 Cr(VI) removal under anaerobic conditions

The ability of the mixed communities in the three sampled mats to remove 1 mg L^-1^ Cr(VI) under anaerobic conditions was primarily tested. The experiments were performed in 160 mL culture bottles, each received 30 mL filtered sterile site water and 0.5 g of homogenized mat. To preserve the native anaerobic bacteria, the anoxic layer of the mat (ca. 2–3 mm thick) was carefully separated using a sterile scalpel, cut into small pieces, and transferred to the bottles within 1–2 min. The anaerobic conditions were created by degassing the culture medium and the headspace in the bottles with pure nitrogen gas (99.9% purity) for 15 min, after sealing the bottles air-tight with rubber stoppers and closing them tightly with aluminum crimps (20 mm) to avoid any air exchange or gas leakage. The bottles were then wrapped with aluminum foil, and incubated in the dark at 30 °C (average air temperature in the sampling site). The bottles were gently shaken at 120 rpm to ensure uniform distribution of nutrients, electron donors/acceptors and microorganisms within the mat slurry. To make sure that this procedure created strictly anaerobic conditions throughout the experiment, methylene blue was used into a separate bottle, prepared identically but without mats. This indicator was not added to any experimental bottles. The color of methylene blue turned colorless, indicating absence of oxygen.

Since all three mats demonstrated similar Cr(VI) removal at the concentration of 1 mg L^-1^ Cr(VI), we randomly chose Mat A to test its ability to remove Cr(VI) at higher concentrations. Fresh original samples of Mat A were then incubated under the same conditions as previously mentioned under varying Cr(VI) concentrations of 5, 15, 20, and 50 mg L^-1^ for 45 days depending on the specific Cr(VI) concentrations. Cr(VI) removal was monitored at different time intervals in all these incubations. Samples were collected and centrifuged at 5,000 rpm for 10 min, and the concentration of Cr(VI) in the supernatant was determined using a ready-made kit provided by TRACE–HT22 chromium hexavalent batch code R09A. The removal of Cr(VI) was evaluated by calorimetrically analyzing the color complex developed by binding of Cr(VI) with diphenyl carbazide (0.03%) under acidic conditions at 540 nm according to EPA7196A (US Environmental Protection Agency, 1992).

### 2.3 Mechanism of anaerobic Cr(VI) removal

A new series of incubations was conducted using only Mat A to elucidate the underlying mechanism of Cr(VI) removal by the mat’s mixed bacterial communities under anaerobic conditions. A mat slurry was prepared by cutting the anoxic mat layer (2–3 mm) into small pieces in sterile site water using a sterile scalpel. An equal volume of the slurry (ca. 200 μL) was added into 160 mL autoclaved bottles containing 30 mL site water amended with 1, 10, 50, or 100 mg L^-1^ Cr(VI) under the same anaerobic conditions described above. The bottles were incubated for 30 days. The site water with Cr(VI), but without mat slurry served as a control. At the end of the incubations, the following analyses were conducted to study the different Cr(VI) removal mechanisms:-

*Biosorption*: To confirm the biosorption of Cr(VI) on the surface of Mat A, scanning electron microscopy with energy dispersive X-ray spectrometry (SEM-EDX) (EDX: Jeol JSM-7,600, United States) analysis was performed. The mat sample was prepared by adding Karnovosky fixative buffer and incubation at 4 °C for 4 h. At the end of the incubation, the fixative buffer was removed, and the mat was washed using different concentrations of ethanol (100%, 90%, 70% and 50%). The sample was then dried at room temperature, fixed on aluminum stubs, platinum coated in vacuum, and analyzed using SEM. Elemental analysis using mapping by SEM-EDX was performed for Cr. To obtain information about the influence of metal ions on surface alterations and the functional groups involved in biosorption, fourier-transform infrared spectroscopy (FTIR) (FT-IR ALPHA II Platinum ATR, Bruker, Germany) analysis was conducted on mat pieces incubated with and without Cr(VI). The mat biomass was collected by centrifugation at 5,000 rpm for 10 min, dried at 80 °C for 24 h and then submitted for FTIR analysis at Sultan Qaboos University Central Laboratory (CAARU). The production of EPS, which are known to facilitate Cr biosorption, by the mat was determined in the absence (control) and in presence of 100 mg L^-1^ Cr(VI). EPS was extracted from the mats using formaldehyde (36.5%) and TCA (20% w/v), and quantified by weighing its amount after drying (Bales et al., 2013).

*Bioreduction*: The whole bottle content was centrifuged at 5,000 rpm for 10 min, and total Cr, Cr(VI), and Cr(III) were determined in the supernatant and in the mat biomass. The mat biomass was subjected to digestion using 1% HNO_3_ before analysis. Total Cr content was determined using a combination of techniques including inductively coupled plasma-optical emission spectrometry (ICP-OES) (Optima 8000DV, Perkin Elmer, United States), whereas Cr(VI) concentrations were measured using the aforementioned method. Cr(III) was then calculated by deducting the amount of Cr(VI) estimated colorimetrically from the total amount of Cr determined by ICP-OES (Khan et al., 2024).

To assess the activity of chromate reductase enzyme, the mat biomass was placed in an ice bath, and the cells were disrupted by ultrasonication (Bandelin, DT 510 H, Germany). The sonicate was then centrifuged and filtered (0.22 µm) to produce cell-free extract (CFE). The optimum contact time that is required to remove 1 mg L^-1^ of Cr(VI) using 1 mL of CFE was first determined, and was found to be 30 min. The enzyme assay was performed by adding 0.2 mL of CFE to a pre-incubated (at 30 °C for 5 min) reaction mixture that contained 1 mg L^−1^ Cr(VI) in 0.8 mL of 100 mM phosphate buffer, pH 7.0. Cr(VI) reduction was then measured after 30 min. One unit of enzyme activity was defined as the amount of enzyme that reduced 1 µmol Cr(VI) min^−1^ at 30 °C as described previously (Camargo et al., 2003).

### 2.4 Bacterial community shifts

MiSeq amplicon sequencing was performed on both the original mat samples (i.e., Mat A, B and C), analyzed immediately after sampling, and on the incubated Mat A samples with and without Cr(VI) to determine the shifts in the bacterial community composition in response to the incubation at different concentrations of Cr(VI). Genomic DNA was extracted from triplicate mat samples using the skim milk method (Volossiouk et al., 1995). The pure DNA extracts were sent to Molecular Research MR DNA laboratory (www.mrdnalab.com, Shallowater, TX, United States) to carry out paired-end Illumina MiSeq sequencing of the bacterial 16S rRNA genes. The primers 341F (5′-CCTACGGGNGGCWGCAG-3′) and 805R (5′-GACTACHVGGGTATCTAATCC-3′) were used, with barcodes incorporated on the forward primer (Klindworth et al., 2013). PCR amplification employed the HotStarTaq Plus Master Mix Kit (Qiagen, United States), and the PCR products were checked for their intensity and size by electrophoresis on 2% agarose gel. PCR products were combined and purified using AMPure XP beads. The pooled samples were then used to construct a DNA library in accordance with Illumina’s standard procedures. Sequencing was performed on the MiSeq platform per manufacturer protocols. Sequence processing was handled using the MR DNA pipeline, which included merging sequences, removing barcodes, and excluding sequences under 150 bp or containing ambiguous bases. Further steps involved denoising, chimera elimination, and OTU clustering at 97% similarity. Taxonomic assignments were carried out using BLASTn against curated RDPII and NCBI databases.

### 2.5 Statistical analysis

R software version 4.4.2 was used to run all statistical analyses (R Development Core Team, 2024). OTUs richness and Chao 1 index were calculated based on equal subsets of sequences for all samples (to the lowest number of sequences found in any sample), using an R-customized script. Non-metric multidimensional scaling (NMDS), based on Bray-Curtis dissimilarities of relative sequence abundances of OTUs, was conducted to visualize shifts in bacterial community composition with changing Cr(VI) concentration. The degree of separation (R value) between different clusters was assessed using Analysis of similarity (ANOSIM), performed using PAST program (Clarke, 1993; Hammer et al., 2001). The effect of different Cr(VI) concentrations on OTUs and classes was analyzed using one-way ANOVA by R package packages “*stats*” followed by *post hoc* evaluation using Tukey’s multiple comparison test between different Cr(VI) concentrations for the variables that showed significant differences between treatments at 0.05 threshold level in all cases using R package packages “*emmeans*” and “*multcomp*” (Hothorn et al., 2008; Lenth, 2025). Moreover, correlations between bacterial classes and different Cr(VI) concentrations as well as most dominant OTUs and Cr(VI) concentrations were run by calculating the Pearson correlation coefficient (r) using R package “*corrplot*” version 0.95. This correlation analysis will explore the potential relationships between detected bacterial classes/OTUs and Cr(VI) concentrations, as well as co-occurrence patterns among taxa, which may point to shared ecological niches or syntrophic interactions under Cr(VI) stress.

## 3 Results

### 3.1 Anaerobic Cr(VI) removal by microbial mats

**FIGURE 1:**
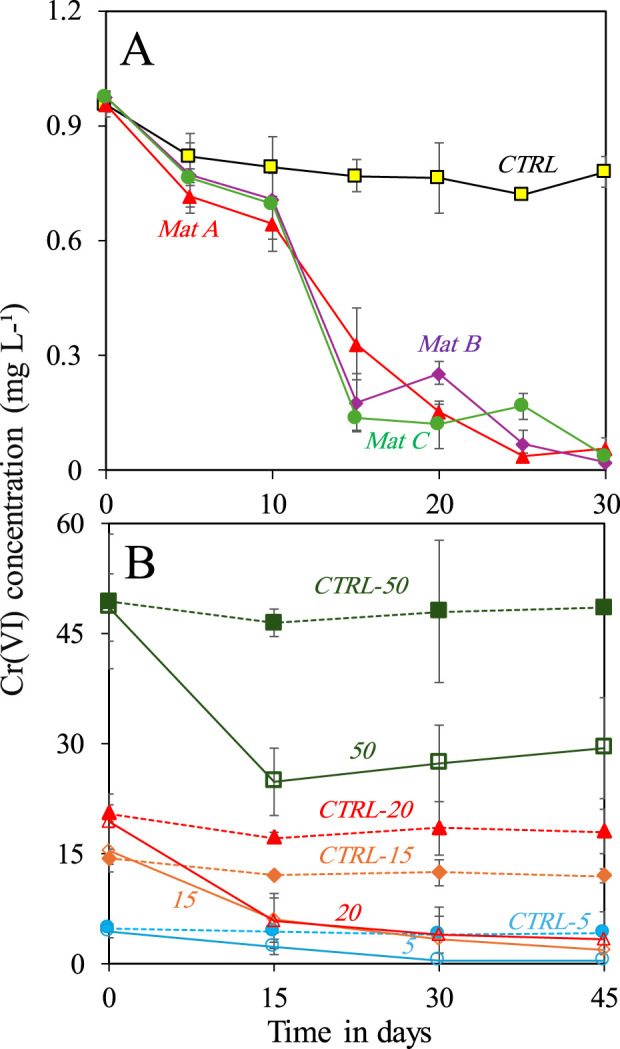
**(A)** Removal of 1 mg L^-1^ Cr(VI) by three microbial mats (A, B and C) collected from Nakhal quarry sumps after incubation in the dark under anaerobic conditions, and **(B)** removal of different concentrations (5, 15, 20 and 50 mg L^-1^) of Cr(VI) by one selected mat (Mat A) under anaerobic conditions. Each incubation was done in triplicates (*n* = 3). Error bars represent ±standard deviation.

Mat A, B, and C removed ≥96 ± 2% of 1 mg L^-1^ of Cr(VI) after incubation for 30 days under anaerobic conditions, as confirmed by Cr(VI) colorimetric analysis (Figure 1A). The Cr(VI) removal rates by the three mats were comparable and reached an average of 0.03 ± 0.001 mg L^-1^ d^-1^. The controls showed minor changes in Cr(VI) concentration over the same period. Further experiments with Mat A at different Cr(VI) concentrations showed an increase in the amount of Cr(VI) removed from the medium with increasing Cr(VI) concentrations (Figure 1B). For instance, Mat A removed 4.7 ± 0.15 mg L^-1^ (corresponding to 94% ± 3% of initial concentration) and 13.9 ± 0.45 mg L^-1^ (corresponding to 93% ± 3% of initial concentration) of Cr(VI) when incubated at the concentrations of 5 and 15 mg L^-1^ Cr(VI) for 45 days, respectively (Figure 1B). Most of Cr(VI) removal occurred in the first 30 days of incubation. When the mat was incubated at 20 mg L^-1^ Cr(VI), approximately 13 ± 0.6 mg L^-1^ (ca. 65% ± 3% of initial concentration) were removed after 45 days. At 50 mg L^-1^, approximately 43% ± 5% of the initial concentration (21 ± 2.5 mg L^-1^) of Cr(VI) was already removed after 15 days of incubations, after which not much removal was observed (Figure 1B). The Cr(VI) removal rates calculated after 30 days for all treatments increased from 0.15 ± 0.01 to 0.77 ± 0.05 mg L^-1^ d^-1^ when the mats was incubated at 5 mg L^-1^ and 50 mg L^-1^ of Cr(VI), respectively.

### 3.2 Biosorption of Cr(VI) under anaerobic conditions

**FIGURE 2:**
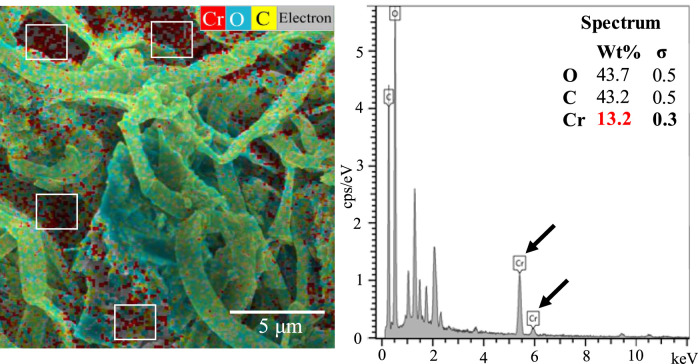
SEM-EDX with elemental mapping of Mat A incubated in the presence of 100 mg L^-1^ of Cr(VI) at 30 °C. Data presented are the averages of three replicates.

**FIGURE 3:**
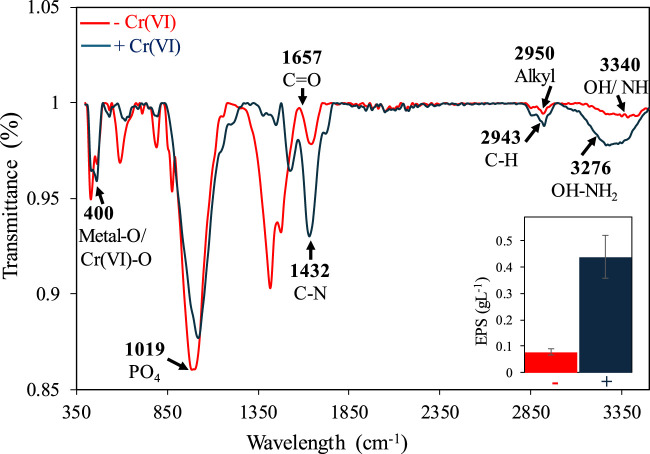
FTIR analysis of Mat A incubated with and without 100 mg L^-1^ of Cr(VI). The arrows indicate the functional groups that exhibited shifts after exposure to Cr(VI). The inserts show increased EPS production upon exposure to Cr(VI).

Biosorption of Cr on the mat’s surface was confirmed using SEM-EDX combined with elemental mapping. SEM-EDX analysis revealed the presence of Cr primarily on the upper layer of Mat A, which was further corroborated by elemental mapping (Figure 2). The data obtained by SEM-EDX for biosorption of Cr(VI) was further validated by FTIR, which identified the key functional groups involved in the process (Figure 3). It was found that metal-O, Cr(VI)-O, PO_4_, C-N, C=O, C-H, Alkyl, and OH-NH_2_ were the main functional groups (Figure 3), as indicated by respective changes in the wavelength of raw biomass and biomass loaded with Cr(VI). The alternations in the wavelengths occurred specifically at 400, 1,019, 1,432, 1,657, 2,943, 2,950, 3,276, and 3,340 cm^-1^ (Figure 3). Additionally, EPS analysis showed approximately three-fold increase in EPS secretion in Mat A upon exposure to Cr(VI) 100 mg L^-1^ under anaerobic conditions (insert in Figure 3). The amount of Cr(VI) adsorbed on the mat’s surface reached 0.32 mg L^-1^ when the mat was incubated in 1 mg L^-1^ Cr(VI), but increased with increasing concentration to reach 31 mg L^-1^ at 100 mg L^-1^ incubation (Table 1).

**TABLE 1:** Removal of Cr(VI) at different concentrations (mg L^-1^) by a microbial mat through bioreduction to Cr(III) and biosorption on cell surface.

Cr(VI) concentration	Remaining Cr(VI) in the supernatant	Bioreduction Cr(VI) to Cr(III)		Total bioreduction	Biosorption in the mat
		Supernatant	Mat		
1	0.02	0.44	0.22	0.66	0.32
10	0.03	2.32	0.95	3.27	6.70
50	20.01	1.19	11.00	12.19	17.80
100	22.00	3.00	44.00	47.00	31.00

### 3.3 Bioreduction of Cr(VI) under anaerobic conditions

**FIGURE 4:**
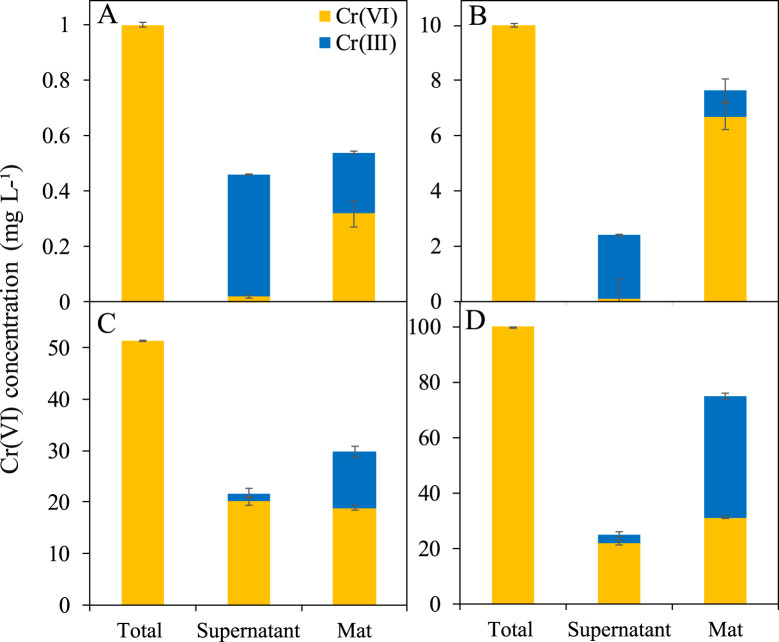
Bioreduction of Cr(VI) to Cr(III) in Mat A incubated at **(A)** 1 mg L^-1^, **(B)** 10 mg L^-1^, **(C)** 50 mg L^-1^, and **(D)** 100 mg L^-1^ calculated by subtracting the colorimetrically estimated amount of Cr(VI) from the total Cr measured using ICP-OES. Each mat was incubated in triplicates. Error bars represent ±standard deviation (*n* = 3).

Bioreduction of Cr(VI) was evaluated by analyzing the concentration of total Cr, Cr(VI), and Cr(III) in the supernatant and in the mat after acid digestion (Figure 4; Table 1). At low Cr(VI) concentrations (1 and 10 mg L^-1^), Mat A reduced more Cr(VI) to Cr(III) in the supernatant than in the mat (Figures 4A,B). At 1 mg L^-1^ Cr(VI), 0.44 mg L^-1^ was converted to Cr(III) in the supernatant (44% of initial concentration), whereas only 0.22 mg L^-1^ (22% of initial concentration) was reduced in the mat (Figure 4A; Table 1). At 10 mg L^-1^ Cr(VI), 2.32 mg L^-1^ was converted to Cr(III) in the supernatant (23.2% of initial concentration), but only 0.95 mg L^-1^ (9.5%) in the mat (Figure 4B; Table 1). At higher Cr(VI) concentrations (50 and 100 mg L^-1^), most of the Cr(VI) was reduced to Cr(III) in the mat than in the supernatant (Figures 4C,D). In the supernatant, ≤3 mg L^-1^ of Cr(III) was detected in both cases. In the mat, ca. 29 and 75 mg L^-1^ of the total Cr was detected at 50 and 100 mg L^-1^, respectively, with 11 and 44 mg L^-1^ of this being in the form of Cr(III) (Figures 4C,D; Table 1). This corresponds to ca. 58% and 75% Cr(VI) reduction to Cr(III), respectively. Overall, the total reduction of Cr(VI) to Cr(III) by Mat A increased with increasing concentration from 0.66 mg L^-1^ when the mat was incubated at 1 mg L^-1^47 mg L^-1^ when the mat was incubated at 100 mg L^-1^ of Cr(VI) under anaerobic conditions (Table 1). The crude extract enzyme (CFE) from Mat A contained a total protein of 3.3 g L^-1^, and a calculated specific activity of 0.034 units (U) mg^-1^ of protein. CFE could successfully remove 80% of 1 mg L^-1^ of Cr(VI) using 0.1 U of the enzyme (CFE).

### 3.4 Shifts in bacterial communities

MiSeq analysis of the original mats revealed the dominance of Proteobacteria, Bacilli, Phycisphaerae, Chloroflexia, Actinobacteria, Clostridia, and Deinococci (Supplementary Figure S2). In Mat A, Bacilli and Phycisphaerae dominated the replicate A1, while Chloroflexia dominated the replicates A2 and A3. Mat B showed high abundance of Bacilli in the replicates B2 and B3, and Actinobacteria, Bacilli, and Clostridia in the replicate B1. In Mat C, Deinococci dominated the replicate C1, whereas Actinobacteria and Proteobacteria were prevalent in the replicates C2 and C3 (Supplementary Figure S2). Among the most dominant genera detected in the mats were *Desulfovibrio*, *Desulfomicrobium*, *Fusibacter*, *Desulfomaculum*, *Bacillus*, *Paenibacillus*, and *Arthrobacter* (Supplementary Figure S2).

**FIGURE 5:**
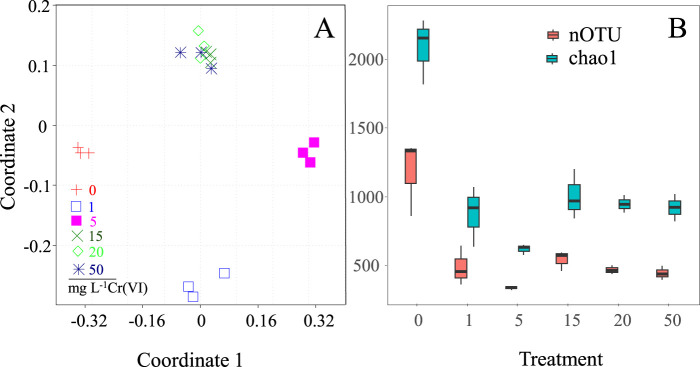
**(A)** NMDS ordination plot using Bray-Curtis dissimilarity showing the shifts of the mat’s bacterial communities after exposure to different concentrations (1, 5, 15, 20 and 50 mg L^-1^) of Cr(VI) **(A)**, and **(B)** estimated OTU richness and Chao1, calculated based on equal subsets of sequences for all samples (to the lowest number of sequences found in any sample), at each Cr(VI) concentration.

Non-metric multidimensional scaling (NMDS) analysis of Mat A incubated with and without Cr(VI) for 45 days showed a clear segregation of bacterial communities into clusters based on the Cr(VI) concentration to which the mat was exposed (Figure 5A). The exposure of Mat A to 1 mg L^-1^ and 5 mg L^-1^ Cr(VI) induced noticeable significant change in the bacterial community structure, resulting in the formation of two distinct clusters (Figure 5A). This separation was supported by the analysis of similarity (ANOSIM), based on Bray-Curtis dissimilarity, that gave an R value of >0.95. Higher Cr(VI) concentrations (15–50 mg L^-1^) caused a further change in the bacterial community composition, leading to the formation of an additional separate cluster (Figure 5A). This cluster was significantly distinct from the other two clusters (ANOSIM R ≥0.85, P = 0.01). The bacterial diversity, indicated by nOTU and Chao, decreased significantly after exposure of the mat to Cr(VI) (P < 0.001 and <0.001, *F* = 5.08 and 5.26 for nOTU and chao1 respectively, Figure 5B).

**FIGURE 6:**
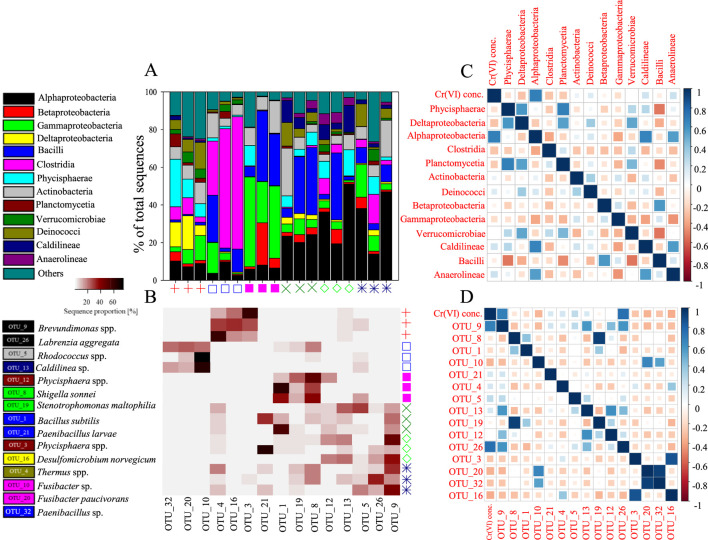
**(A)** The relative abundance (%) of major phyla and classes of bacteria present in the mat incubated with and without Cr(VI). The symbols identifying the different mat samples are the same as in Figure 5. **(B)** A heatmap showing the relative abundance (%) of the most dominant OTUs detected after incubation with and without Cr(VI), **(C)** A plot showing the extent of correlation of different bacterial classes/phyla with Cr(VI) concentration and correlation between bacterial classes/phyla in pairwise comparison as determined by the Pearson correlation coefficient (r), and **(D)** Correlation plot between bacterial OTUs and Cr(VI) concentration and between bacterial classes/phyla in pairwise comparison as determined by the Pearson correlation coefficient.

MiSeq analysis of the Cr(VI)-free mats revealed that Phycisphaerae, Deltaproteobacteria and Deinococci were the dominant classes in each of the replicate samples, accounting for 25%, 18% and 14% of the total sequences, respectively (Figure 6A). The main OTUs encountered belonged to the Phycisphaerae-related genus *Phycisphaerae*, Deltaproteobacterial genus *Desulfomicrobium*, and Deinococci-related genus *Thermus*. Alpha-, Gamma-proteobacteria and Actinobacteria were also detected at ≤13% relative abundances. The incubation of Mat A in 1 mg L^-1^ Cr(VI) resulted in a prominent shift in the bacterial community structure in favor of Clostridia and Bacilli, which constituted between 54%–82% of total sequences in each of the replicate samples (Figure 6A). The most prevalent clostridia-related OTUs (OTU_10 and OTU_20) were affiliated to *Fusibacteria* species, whereas the most prevalent Bacilli-related OTU was affiliated to *Paenibacillus* species (Figure 6B). When Mat A was exposed to 5 mg L^-1^ Cr(VI), the relative abundance of sequences related to Gammaproteobacteria increased to make up between 22% and 48% of total sequences in each replicate sample (Figure 6A). The most prevalent OTUs detected in this group were related to *Shigella sonnei* (OTU_8), and *Stenotrophomonas maltophilia* (OTU_19) (Figure 6B). The sequence proportion of Bacilli at 5 mg L^-1^ Cr(VI) treatment also increased, at least in two replicates, whereas the proportion of Betaproteobacteria sequences increased in one replicate (Figure 6A). The most dominant OTU among the bacilli sequences (OTU_1) was related to *Bacillus subtilis* (Figure 6B).

When Mat A was incubated at higher Cr(VI) concentrations, between 15 and 50 mg L^-1^, all samples exhibited a dominance of Alphaproteobacteria (Figure 6A). The relative abundance of this group reached between 14% and 51% of total sequences in each sample, with OTUs (OTU_9 and OTU_26) related mainly to *Brevundimonas* and *Labrenzia* species (Figure 6B). Bacilli continued to persist in two replicate samples of the mat exposed to 15 mg L^-1^ Cr(VI), but only in one replicate of the mat exposed to 20 mg L^-1^ Cr(VI) (Figure 6A). The relative abundance of Bacilli in these samples reached between 30% and 36%, each of total sequences. At 50 mg L^-1^ Cr(VI), the relative abundance of Bacilli decreased to reach ≤9% of total sequences. Actinobacteria made up to 25% of total sequences in only one sample incubated at 15 mg L^-1^ Cr(VI), and 20% in another incubated at 50 mg L^-1^ Cr(VI) (Figure 6A). Sequences belonging to Actinobacteria in these two samples were mostly affiliated to *Rhodococcus* species (Figure 6B). While bacterial groups such as Caldilineae and Phycisphaerae were detected in one of the samples incubated at 20 mg L^-1^ Cr(VI), each at a relative abundance of 14% of total sequences, other groups such as Gammaproteobacteria, Clostridia and Deinococci appeared again at least in one of the samples incubated at 50 mg L^-1^ Cr(VI) at a relative abundance between 12% and 17%.

A significant positive correlation was observed between Alphaproteobacteria and the different Cr(VI) concentrations (Pearson r = 0.75, p < 0.001, Figure 6C). Alphaproteobacteria also showed positive correlations with Caldilineae (r = 0.66, p = 0.002) and Anaerolineae (r = 0.55, p = 0.01, Figure 6C). Bacilli exhibited negative correlations with Phycisphaerae (r = −0.52, p = 0.02), Planctomycetia (r = −0.47, p = 0.04), and Verrucomicrobiae (r = −0.53, p = 0.02). Phycisphaerae correlated positively with Deltaproteobacteria (r = 0.58, p = 0.01) and Planctomycetia (r = 0.68, p = 0.001). Cr(VI) concentration was positively correlated with OTU_9 (*Brevundimonas*-related; r = 0.75, p < 0.001) and OTU_26 (*Fusibacteria*-related; r = 0.72, p < 0.001; Figure 6D).

## 4 Discussion

### 4.1 Anaerobic Cr(VI) removal rates by microbial mats

Our data demonstrate that microbial mats can effectively remove Cr(VI) under anaerobic conditions at concentrations ranging from 1 to 100 mg L^-1^. The ability of Mat A to remove 78% of 100 mg L^-1^ Cr(VI) in 30 days indicates both tolerance and removal efficiency. We speculate that the mat could tolerate and remove even higher Cr(VI) concentrations with extended incubation time. Cr(VI) removal rates by Mat A increased from 0.03 ± 0.01 to 0.77 ± 0.05 mg L^-1^ d^-1^ as Cr(VI) concentration increased from 1 to 100 mg L^-1^, comparable to rates (0.19–0.56 mg L^-1^ d^-1^) reported for anaerobic bacterial consortia (Martins et al., 2010; Mamais et al., 2016), though generally lower than rates (8.33 and 4.16 mg L^-1^ d^-1^) observed with individual bacterial strains such as *Exiguobacterium* sp. PY14 and *Sporosarcina saromenis* (Huang et al., 2021; Huang et al., 2023). As expected, removal rates were notably slower than under aerobic conditions (i.e. 0.14 ± 0.01 mg L^-1^ d^-1^ at 1 mg L^-1^ Cr(VI)) (Abed et al., 2020), consistent with the generally slower nature of anaerobic processes (Deo et al., 2024). While mixed bacterial communities are generally considered more effective in heavy metal removal than individual strains, due to their diverse metabolic capabilities and synergistic interactions, removal could be affected by competition among bacteria, electron donor availability, and nutrient limitations (Qiang et al., 2013; Qin and Wang, 2017).

### 4.2 Biosorption of Cr(VI) on mat’s surface

Our data demonstrated that Cr(VI) removal under anaerobic conditions by the mat slurry involved biosorption on cell surface. While it was previously postulated that bioadsorption becomes the main process at higher (≥50 mg L^-1^) concentrations (Durán et al., 2018), our data suggested that bioadsorption increased with increasing Cr(VI) concentration. SEM-EDX and elemental mapping data supports the accumulation of Cr(VI) on the surface of the mat biomass, an observation that is consistent with previous findings (Batool, 2012; Khan et al., 2024). Previous studies have demonstrated a vital role of the same functional groups detected by FTIR in the removal of heavy metals including Cr (Shukla et al., 2012; Plaza-Cazón et al., 2022; Khan et al., 2024). In fact, the shifts of the peaks of these functional groups after exposure to Cr(VI) indicates a crucial role of EPS in the removal of Cr(VI), a conclusion further supported by the observed threefold increase of EPS production under Cr(VI) stress. Hence, Cr removal is not merely a physical process but is actively facilitated by microbes through the secretion of EPS. While some studies have shown an increase in EPS production upon exposure to Cr(VI), others have shown an inhibition when Cr(VI) concentrations reached 50 mg L^-1^ (Liu et al., 2017; Khan et al., 2024). In our case, the continuous increase in the adsorbed amount of Cr(VI) with increasing Cr(VI) concentration up to 100 mg L^-1^ suggests a continued EPS production. EPS is known to increase the binding of heavy metals including Cr on cell surface, due to its hydrated, gel-like matrix, and the presence of negatively charged functional groups such as hydroxyl, carboxyl, and amino groups (Shukla et al., 2012; Mathivanan et al., 2021; Pagliaccia et al., 2022; Plaza-Cazón et al., 2022; Khan et al., 2024). EPS extracted from activated sludge was shown to efficiently adsorb ca. 51% of Cr(VI) with the involvement of hydroxyl, carboxyl and amine groups (Liu et al., 2017), whereas algae-derived EPS could remove ca. 237 mg g^-1^ Cr(VI) with the involvement of the same groups (Wu et al., 2018). Cyanobacteria such as *Chlorella* sp., *Phormidium* sp., and *Oscillatoria* sp. from a cyanobacterial mat have demonstrated the ability to remove Cr(VI) with involvement of functional groups such as OH, COO, CO and Cr(VI)-O present in the EPS (Shukla et al., 2012). EPS is also known to contain carbohydrates such as sucrose, maltose and galactose, which could act as a carbon source and an electron donor for Cr(VI) removal under anaerobic conditions (Ehrlich, 1997; Bales et al., 2013). It should also be kept in mind that the quantity and composition of EPS may differ under aerobic and anaerobic conditions, due to different bacterial activities, which could influence Cr(VI) removal, however, this requires further investigations.

### 4.3 Cr(VI) bioreduction under anaerobic conditions

The detection of Cr(III) in both the supernatant and mat suggests that Cr(VI) bioreduction occurred under anaerobic conditions in Mat A. In the absence of oxygen, Cr(VI) acts as electron acceptor, however the exact mechanisms involved remain difficult to elucidate due to the metabolic complexity and diversity of mat microbial communities. Both direct enzymatic reduction of Cr(VI) to Cr(III), mediated by soluble and membrane-bound chromate reductases (Thatoi et al., 2014), or indirect reduction via redox active metabolites such as Fe(II) or H_2_S are plausible pathways. Previous studies have reported that bacteria like *Pantoea agglomerans* SP1 and *Enterobacter cloacae* HO1 performed dissimilatory Cr(VI) reduction to Cr(III) under anaerobic conditions using membrane associated enzyme like flavin reductases, cytochromes and hydrogenases (Thatoi et al., 2014). *Geobacter Sulfurreducer* PCA also reduced Cr(VI) anaerobically using both intracellular and extracellular enzymes (Gong et al., 2018). Although bioreduction was expected to decrease with increasing concentrations of Cr(VI), due to the toxicity of Cr(VI), the inability of chromate reductase enzyme to reach Cr(VI) trapped inside the mat, and the preoccupation of active sites on the enzyme (Thatoi et al., 2014; Durán et al., 2018; Rahman et al., 2023), it continued to increase with increasing concentrations up to 100 mg L^-1^. This demonstrates a higher efficiency of our mat in removal of Cr(VI) under anaerobic conditions. Thus, microbial mats constitute a promising ecosystem for the bioremediation of Cr-impacted sites, leveraging to presence of diverse types of aerobic and anaerobic microorganisms, and the availability of different organic carbon sources as photosynthetic and fermentation products, that can act as electron donor for Cr(VI) bioreduction. However, more research is still required to find out the precise biochemical and molecular mechanisms underlying Cr(VI) bioreduction, which is crucial for optimizing bioremediation strategies.

### 4.4 Bacterial community shifts

The formation of separate clusters in the NMDS analysis at Cr(VI) concentrations of ≤5 mg L^-1^ and ≥15 mg L^-1^ suggests the existence of microorganisms with different Cr(VI) tolerance levels within the investigated mat. In fact, the prominent decrease in OTU richness and Chao index after exposure to Cr(VI) could be due to the toxic effect on microorganisms in the original mat. This is plausible given that total Cr concentrations in source water where the mats were collected did not exceed 50 μg L^-1^ (Supplementary Table S1). Previous studies have indeed demonstrated an adverse effect of high levels of Cr(VI) on microorganisms by impairing microbial growth and metabolism through the production of reactive oxygen species (ROS) and causing oxidative stress and by damaging DNA leading to mutations (Narayani and Shetty, 2013; DesMarias and Costa, 2019).

The major detected bacterial phyla in the investigated mat; Proteobacteria, Firmicutes and Actinobacteria, have been previously encountered in samples with the ability to remove Cr(VI) (Drewniak et al., 2016; Abed et al., 2020). The enrichment of Clostridia, which contain obligatory anaerobes, and Bacilli, which contain facultative anaerobes indicates a potential active role of these bacteria in the removal of Cr(VI) under anaerobic conditions. Interestingly, there is no direct evidence confirming the presence of chromate reductase enzyme in Clostridia spp., although they are known to possess enzymes that catalyze redox reactions, and there are no known Cr(VI)-reducing isolates belonging to this class. Nevertheless, Firmicutes have been previously detected in several polluted ecosystems, and could play a significant role in the removal of pollutants, including heavy metals, due to their diverse metabolic capabilities (Zheng et al., 2019). For example, *Bacilli*, and *Clostridia* were dominantly detected in an anaerobic hydrogen fermenter and could completely reduce 100 mg L^-1^ of Cr(VI) in 70 h by increasing the production of EPS (Zheng et al., 2019). Anaerobic granular *Clostridia*-dominated consortia could biologically reduce 70 mg L^-1^ of Cr(VI) to Cr III at the concentration of 250 mg L^-1^ Cr (VI) (Durán et al., 2018). On the other hand, facultative anaerobic Bacilli species such as *Bacillus* sp. QH-1, *Bacillus* CRB-1 and *Exiguobacterium* sp. PY14 have been reported for Cr(VI) reduction using chromate reductase enzyme and biosorption using EPS (Xu et al., 2014; Tan et al., 2020; Huang et al., 2023). *B. subtilis* isolated from tannery effluent-contaminated soil was found to grow at 800 mg L^-1^ Cr(VI) and could completely reduce 50 mg L^-1^ in 65 h under anaerobic conditions (Mary Mangaiyarkarasi et al., 2011). The enrichment of sequences belonging to the genera *Fusibacteria* and *Paenibacillus* in Mat A after exposure to Cr(VI) under anaerobic conditions is intriguing as there are very limited studies regarding the involvement of these bacteria in Cr(VI) removal.

The predominance of Gammaproteobacteria in the mat’s bacterial communities at 5 mg L^-1^ suggests a possible role in the anaerobic bioreduction of Cr(VI). Species belonging to Gammaproteobacteria have been previously shown to possess the enzyme chromium reductase and reduce Cr(VI) to Cr(III) under anaerobic conditions (Garavaglia et al., 2010; Baldiris et al., 2018; Raman et al., 2018). For instance, bacterial species isolated from Cr-contaminated industrial waste such as *Pseudomonas veronii* 2E, *Delftia acidovorans* AR, *Klebsiella oxytoca* P2 and *Klebsiella ornithinolytica* 1P could remove Cr(VI) under anaerobic conditions (Garavaglia et al., 2010). Our data showed the enrichment of OTUs belonging mainly to *Stenotrophomonas maltophilia* and *Shigella sonnei*. A previous study has demonstrated the ability of *S. maltophilia* to resist 400 mg mL^-1^ of Cr(VI) (Raman et al., 2018), while another study demonstrated its ability to anaerobically reduce Cr(VI) concentrations ranging from 10 to 500 mg L^-1^ at pH 7 °C and 37 °C using chromate reductase (ChrR) enzyme (Baldiris et al., 2018). On the other hand, there is no research reporting on the role of *Shigella sonnei* in Cr(VI) reduction under anaerobic conditions.

The increase in the relative abundance of Alphaproteobacteria at the concentrations of 15–50 mg L^-1^ Cr(VI) highlights the high tolerance of this group to high Cr(VI) concentrations. Alphaproteobacteria are known to thrive under variable conditions of pH, temperature, and presence of organic matters, which support the reduction of Cr(VI) either via biosorption onto the cell surface or enzymatically using chromate reductase (Narayani and Shetty, 2013; Joutey et al., 2015). This group has been shown to become dominant when the same mat was incubated in the presence of Cr under aerobic conditions, with a noticeable increase in sequences belonging to the genera *Rhizobium* and *Brevundimonas* (Abed et al., 2020). In fact, Cr(VI) tolerant *Brevundimonas* spp. have been detected in several Cr contaminated sites and could reduce up to 350 mg L^-1^ Cr(VI) under aerobic conditions (Lu et al., 2011; Wang et al., 2016). The enrichment of *Brevundimonas* species in the investigated mat under anaerobic conditions suggests that these bacteria belong to facultative anaerobes and were still able to reduce Cr(VI) to Cr(III) in the absence of oxygen. So far, there are no available isolates belonging to this genus with the ability to reduce Cr(VI) under anaerobic conditions. This highlights the vital role of facultative anaerobic bacteria in microbial mats in the removal of heavy metals including Cr(VI). Further investigations are required to isolate representative strains of facultative anaerobes from microbial mats to compare the mechanism(s), optimum conditions, and rates of removal of Cr(VI) under both aerobic and anaerobic conditions.

## 5 Conclusion

We conclude that microbial mats are capable of removing Cr(VI) under anoxic conditions either by EPS-facilitated biosorption onto cell surface, or by enzymatic bioreduction by their obligate and facultative anaerobic bacteria, highlighting their potential for bioremediation of Cr- contaminated environments under low-oxygen conditions. The exact molecular interaction between Cr(VI) and EPS functional groups, changes in the composition and function of EPS by different microbial communities and under different environmental conditions, and the role of EPS in mediating electron transfer during enzymatic reductions remain to be explored. Further studies should focus on isolating anaerobic Cr(VI)-removing mat microorganisms and employ multiomics on isolates, as well as on mixed mat communities, to gain more understanding of metabolic pathways and genetic mechanisms of Cr(VI) removal. Since microbial mats are known to host obligate anaerobes such as sulfate reducing bacteria and methanogens, it is of interest to find out whether such bacteria have the ability to remove Cr(VI) under respective conditions. Exploring the long-term stability and efficiency of microbial mats in Cr(VI) removal could enhance their viability for large-scale bioremediation efforts. Microbial mats can eventually be integrated in engineered bioreactors or natural/constructed wetlands to provide innovative solutions for treating Cr-contaminated anaerobic environments.

## Data Availability

The original contributions presented in the study are publicly available. This data can be found under the BioProject accession number PRJNA1312130.
