# Supplementary material for: Removal of hexavalent chromium by a microbial mat from a mining site under anaerobic conditions
Source: Front Bioeng Biotechnol. 2025 Sep 12;13:1585237. doi: 10.3389/fbioe.2025.1585237 (PMC12463898; doi:10.3389/fbioe.2025.1585237)

## Slide 1
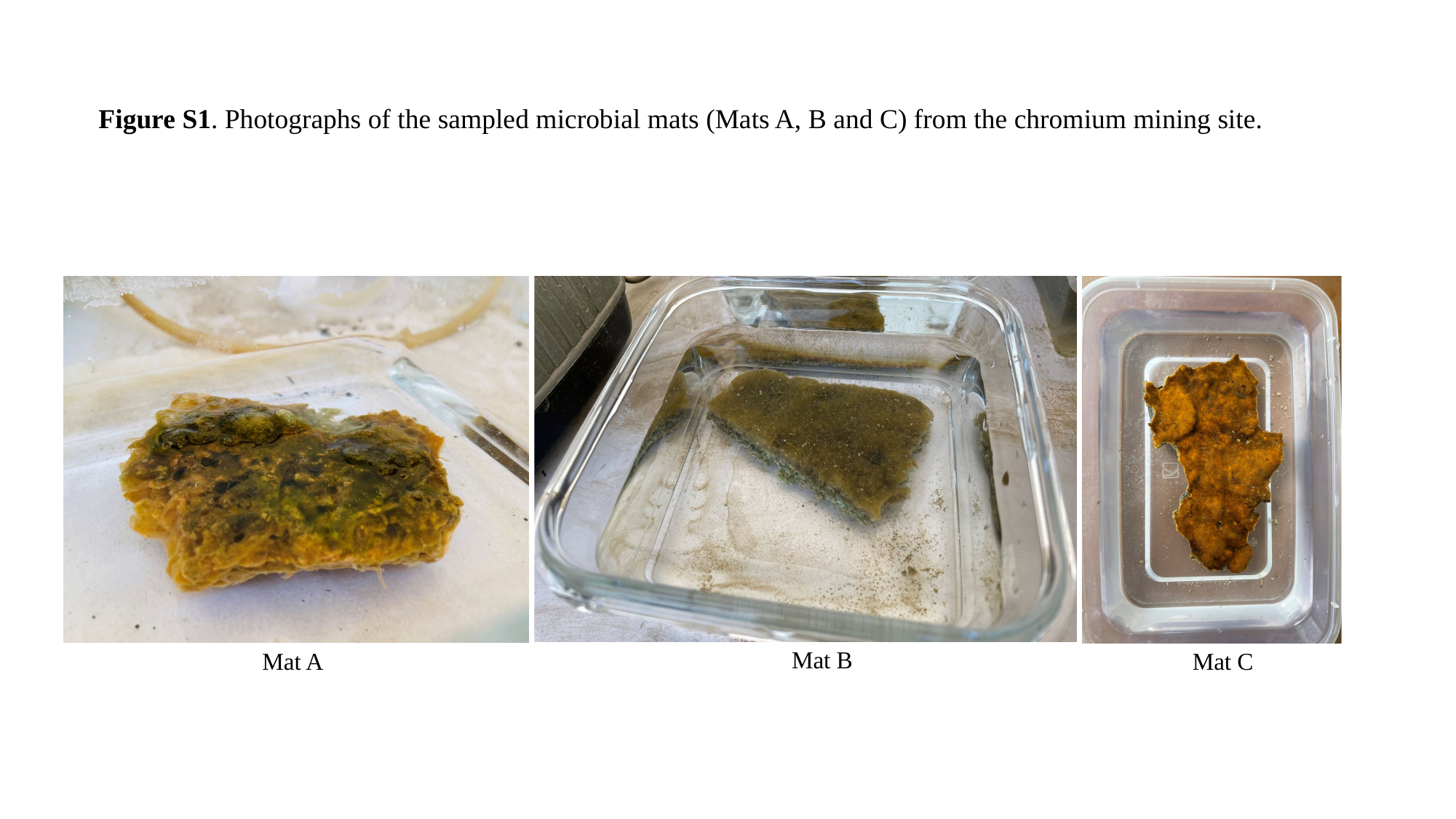

Figure S1. Photographs of the sampled microbial mats (Mats A, B and C) from the chromium mining site.
Mat B
Mat A
Mat C

## Slide 2
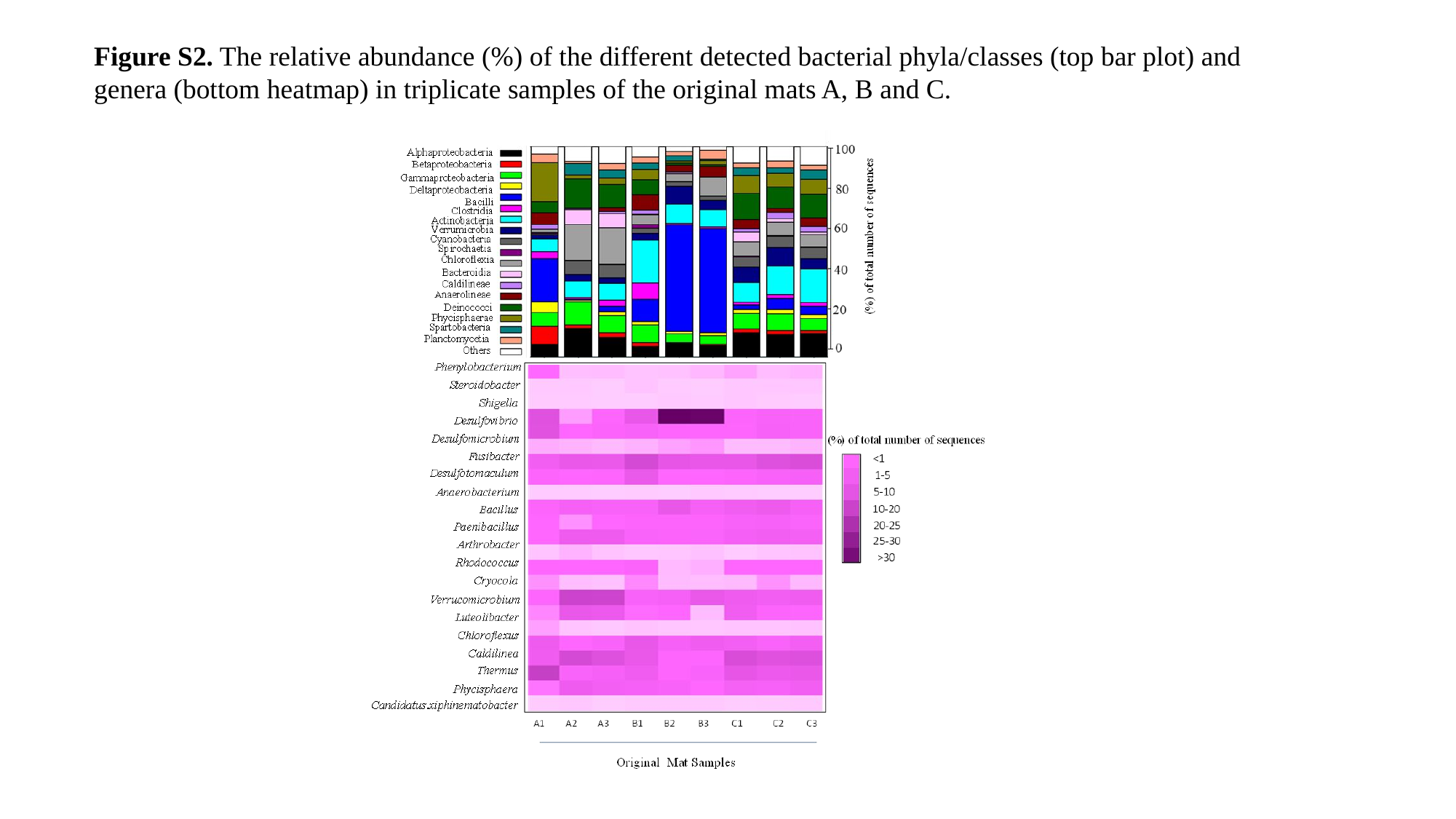

Figure S2. The relative abundance (%) of the different detected bacterial phyla/classes (top bar plot) and genera (bottom heatmap) in triplicate samples of the original mats A, B and C.

## Slide 3
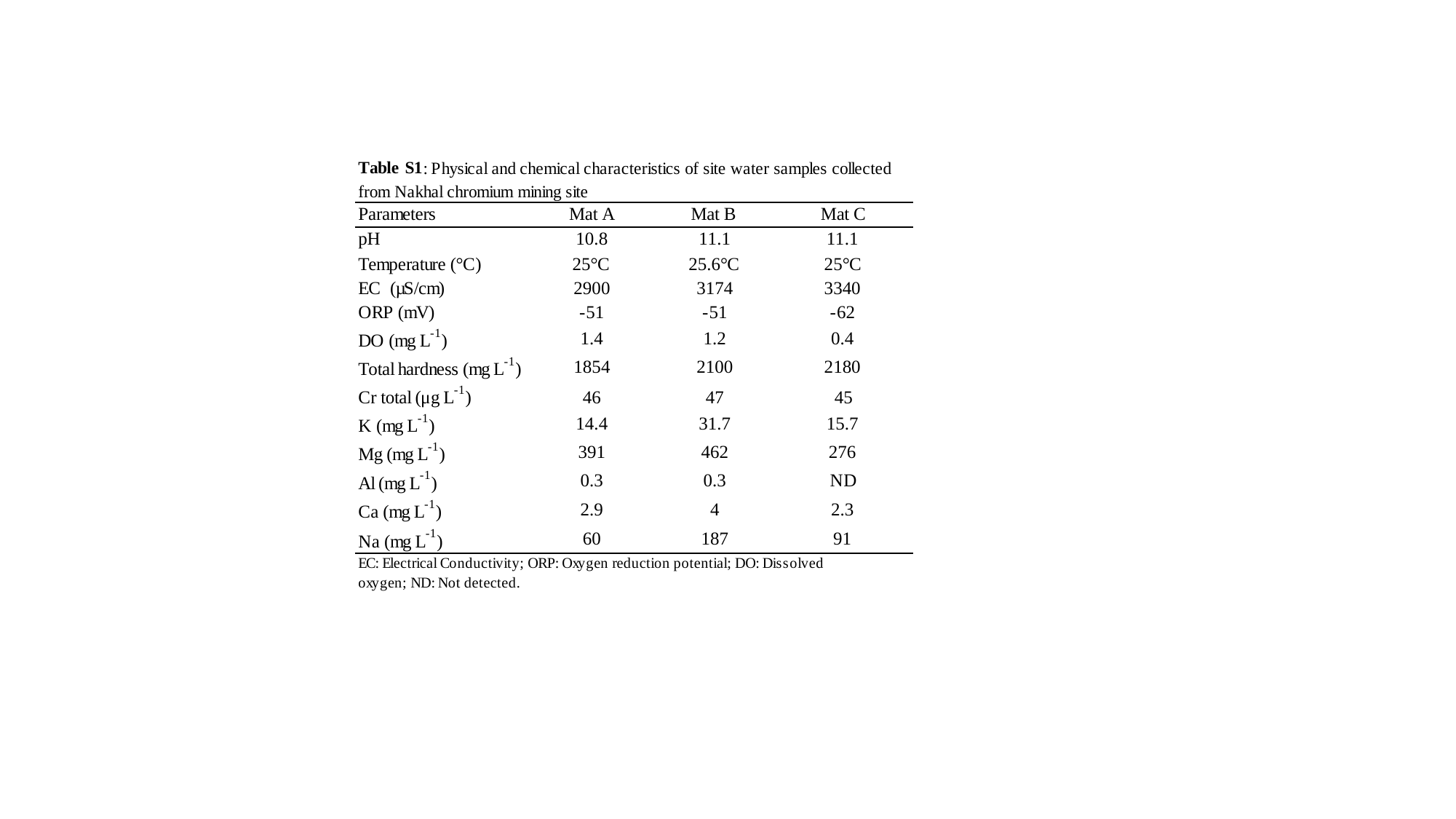

Supplement: Supplementary file 1 [file Presentation1.pptx]
